# Exertional Rhabdomyolysis After CrossFit Exercise

**DOI:** 10.7759/cureus.12630

**Published:** 2021-01-11

**Authors:** Prakash Adhikari, Adithya Hari, Laurent Morel, Yolin Bueno

**Affiliations:** 1 Internal Medicine, Piedmont Athens Regional Medical Center, Athens, USA

**Keywords:** crossfit, rhabdomyolysis, exertional rhabdomyolysis

## Abstract

Multiple studies have reported the metabolic benefits of high-intensity exercise programs like CrossFit. If these high-intensity exercises are not done in a proper structured pattern, adverse outcomes like rhabdomyolysis can occur. Here we discuss a case of a patient who undertook one session of CrossFit exercise and developed exertional rhabdomyolysis. A 22-year-old Caucasian male presented to the emergency department with complaints of generalized body ache and passage of dark-colored urine. His symptoms began after two days of an exhaustive session of CrossFit exercise. Blood test in the emergency showed elevated creatine kinase (CK) of 132,540 units per liter (U/L), normal renal function (creatinine and blood urea nitrogen), and normal serum electrolytes. His clinical symptoms and lab findings were consistent with exertional rhabdomyolysis. He was treated with aggressive intravenous fluids and oral hydration therapy. He did not develop any complication and he was discharged on the sixth day. This case report demonstrates a possible preventable rhabdomyolysis that developed secondary to undue participation in CrossFit exercise.

## Introduction

CrossFit is a form of high-intensity training program that combines aerobic and resistance exercise in short repetitive workout sessions. It was introduced particularly to train individuals whose work requires physical fitness and muscle strength (e.g., police officers, military special forces) [1]. More specifically, it integrates power lifting (squats, dead lifts, bench presses), gymnastics (pull-ups, sit-ups, lunges), and conditioning exercises (swimming, rowing, running), which could make it enjoyable when done with a group-based approach. Since its introduction in early 2000, it has been rapidly adopted by many military and civilian gymnasiums [1]. A systematic review of 13 studies with 2,326 subjects demonstrated numerous cardiometabolic benefits of CrossFit training including increased aerobic capacity, decrease blood pressure, weight loss, and increased insulin sensitivity [2]. However, due to the intense nature of the exercise regime, the risk of injury is also a relevant concern. 

There are several case reports of rhabdomyolysis that developed after CrossFit training [3,4]. Rhabdomyolysis is characterized by the breakdown of muscle tissue and spilling of intramyocellular contents into the systemic circulation. Acute kidney injury (AKI) is the most common and serious complication which is thought to be due to high amount of free myoglobin which causes renal vasoconstriction, nephrotoxic effects, and renal tubular obstruction [5]. Electrolyte imbalance, especially hyperkalemia is another dreaded complication of rhabdomyolysis. Rhabdomyolysis due to strenuous exercise is termed as exertional rhabdomyolysis. The incidence of exertional rhabdomyolysis is estimated to be 29.9/100,000 [6]. It usually presents with generalized muscle soreness, pain, swelling, or stiffness within 24-48 hours after the inciting event. In severe conditions, it can also cause cardiac arrhythmia and even death. We report a case of a healthy patient who presented to our hospital with exertional rhabdomyolysis after an unaccustomed CrossFit exercise.

## Case presentation

A 22-year-old Caucasian male with no significant past medical history presented to the emergency department with complaints of generalized body ache and passage of dark-colored urine for one day. The pain was more severe around his chest, upper back, arms, and shoulders. Two days prior to the onset of symptoms, he participated in CrossFit exercise which included three hours of abdominal crunches, sit-ups, and weight lifting. This was different from his usual routine exercise regimen. The patient did not report the use of over-the-counter supplements, statins, nonsteroidal anti-inflammatory drugs, and alcohol. Physical examination was unremarkable. Initial blood test showed elevated creatine kinase (CK) >20,000 U/L, later the actual value was quantified using diluted method and it was 132,540 U/L. Aspartate aminotransferase (AST, 136 U/L), alanine aminotransferase (ALT, 772 U/L) was elevated but creatinine (1.05 mg/dL) was normal. Urine analysis was positive for blood but red blood cells were not present in the urine. He was diagnosed with rhabdomyolysis based on his clinical features and elevated CK. He was admitted to the hospital. He was treated aggressively with intravenous and oral fluid. The serum CK level decreased gradually. The value of CK for each day is shown in Table 1. He did not develop any other complications including acute renal failure. The serum CK level on the sixth day was 7980 U/L and he was discharged with instructions to avoid sudden strenuous physical activity but to scale up in a graded fashion.

**Table 1:** Quantification of creatine kinase

Days of Admission	Creatine kinase (U/L)
Day 1	132,540
Day 2	116,940
Day 3	67,400
Day 4	16,523
Day 5	15,602
Day 6	7,980

## Discussion

Exertional rhabdomyolysis occurs due to excessive physical activity leading to depletion of local muscle energy store and the inability of muscle cells to maintain cellular integrity. This can happen in healthy individuals and results in cell damage, release of cellular contents, and ultimately secondary complications [5]. Currently, there are no set guidelines in the diagnosis of rhabdomyolysis. CK level greater than five times the upper limit is a common finding in rhabdomyolysis. CK is also found in cardiac tissue and the brain apart from striated muscles. Previous studies have shown a strong positive correlation between peak CK level and length of hospital stay, possibly due to the extent of muscle damage [7]. The level of other skeletal muscle enzymes including ALT and AST also increases in rhabdomyolysis. The most common clinical presentation of rhabdomyolysis is myalgia and dark colored urine, indicating myoglobinuria. AKI is a serious complication. High amounts of free myoglobin causes renal vasoconstriction, nephrotoxic effects, and renal tubular obstruction leading to AKI [5]. There is a positive correlation between myoglobin and CK. CK serves as a cheaper surrogate, so it is commonly used to diagnose rhabdomyolysis. In the study by Terpilowski and Criddle, it has been shown that CK level of 20,000 U/L is the threshold to begin treatment to prevent renal failure with rhabdomyolysis [8].

It is well known that sudden high-intensity exercise can cause exertional rhabdomyolysis. Little exercise experience, eccentric muscle contraction, history of electrolyte abnormality, low protein diet, statin, alcohol use, and creatine supplementation are associated with increased risk for development of rhabdomyolysis [4]. Amidst numerous metabolic benefits, CrossFit training can cause exertional rhabdomyolysis, especially in beginners who do extensive sessions without initial warmup. Our patient was basically a healthy and fit adult with no prior medical condition, and he was not taking any medication. He participated in a CrossFit exercise session after a break of a few weeks from his usual routine and ended up in the hospital with exertional rhabdomyolysis. This case report highlights the possible preventable cause of exertional rhabdomyolysis.

## Conclusions

CrossFit exercise has various health benefits like increased aerobic capacity, decrease in blood pressure, weight loss and increased insulin sensitivity. As with any kind of strenuous exercise, sudden participation in high-intensity CrossFit exercise can result in exertional rhabdomyolysis, requiring hospital admissions. The number of cases of rhabdomyolysis after CrossFit exercise are increasing as CrossFit has been adopted by many gyms and training centers. Exertional rhabdomyolysis due to CrossFit is potentially preventable, if participants and trainers are made aware of complications of undue participation in CrossFit exercise.
